# Advancements in Injectable Hydrogels for Controlled Insulin Delivery: A Comprehensive Review of the Design, Properties and Therapeutic Applications for Diabetes and Its Complications

**DOI:** 10.3390/polym17060780

**Published:** 2025-03-14

**Authors:** Lin Li, Ya Wang

**Affiliations:** Guangdong Provincial/Zhuhai Key Laboratory of IRADS, and Department of Life Sciences, BNU-HKBU United International College, Zhuhai 519087, China; llli12@student.unimelb.edu.au

**Keywords:** injectable hydrogels, controlled insulin delivery, diabetes, diabetic complications

## Abstract

Glycemic management in diabetes patients remains heavily reliant on multiple daily insulin injections, which often leads to poor patient compliance and an elevated risk of hypoglycemia. To overcome these limitations, injectable hydrogels capable of encapsulating insulin within polymeric networks have emerged as a promising alternative. Ideally, a single injection can form an in situ depot that allows prolonged glycemic control and lower injection frequency. This review summarizes recent advances in injectable hydrogels for controlled insulin delivery, focusing on the polymer sources, crosslinking strategies, and stimuli-responsive release mechanisms. Synthetic polymers such as PEG, PNIPAM, and Pluronics dominate the current research due to their highly tunable properties, whereas naturally derived polysaccharides and proteins generally require further modifications for enhanced functionality. The crosslinking types, ranging from relatively weak physical interactions (hydrogen bonds, hydrophobic interactions, etc.) to dynamic covalent bonds with higher binding strength (e.g., Schiff base, phenylboronate ester), significantly influence the shear-thinning behavior and stimuli-responsiveness of hydrogel systems. Hydrogels’ responsiveness to temperature, glucose, pH, and reactive oxygen species has enabled more precise insulin release, offering new options for improved diabetic management. Beyond glycemic regulation, this review also explores insulin-loaded hydrogels for treating complications. Despite the progress, challenges such as burst release, long-term biocompatibility, and scalability remain. Future research should focus on optimizing hydrogel design, supported by robust and comprehensive data.

## 1. Introduction

Diabetes is one of the major challenges to global health. The prevalence of diabetes has continued to rise in recent years and is estimated to exceed 783.2 million in 2045 [1]. As a serious chronic disease, diabetes is characterized by persistent hyperglycemia, which leads to multiple complications, including the loss of kidney function and lower limb amputation [2]. The main types of diabetes mellitus are type 1 and type 2, and insulin therapy is an effective method to regulate abnormal blood glucose levels [3]. The most common administration method for insulin is subcutaneous injection, but it leads to decreased compliance and tissue infections due to the need for multiple daily injections [4]. In the US, over 3.5 million individuals requiring insulin therapy experience poor treatment outcomes. One major contributing factor is fear of insulin injections, arising from needle anxiety, diminished social engagement, and complex regimens. The DARTS/MEMO study confirmed poor adherence to insulin therapy. Nearly 30% of patients with type 1 diabetes used less than the prescribed dose [5]. Severe hypoglycemia occurs occasionally in patients with type 2 diabetes, but its annual incidence can be up to 13.5% in those with type 1 diabetes [6]. Non-invasive treatments such as oral administration and inhalation are promising, but the limitations of poor bioavailability and significant variations in absorption still exist [7]. Injection remains the preferred method for insulin administration, and the key lies in developing controlled and prolonged delivery systems to improve therapeutic efficacy and reduce the frequency of injections and the risk of adverse effects [8]. Meanwhile, considering that diabetes will result in severe complications and that insulin has been indicated to play an important role in controlling the progression of diabetic complications such as retinopathy, neuropathy, and nephropathy, medications that combine insulin with other therapeutic agents, like liraglutide and manganese dioxide nanoenzyme, to improve the treatment effectiveness are also reported [9,10,11,12].

Hydrogels are polymers with porous 3D network structures that can hold a large amount of water, and their physicochemical properties are similar to the extracellular matrix [13]. Hydrogels with biodegradability and biocompatibility can undergo degradation or metabolism in the human body while also providing a suitable environment for cell growth [14]. Moreover, by adjusting the composition, molecular weight, crosslinking type and density, hydrogels’ physical and mechanical properties, capacity to encapsulate pharmaceuticals and stimuli-responsiveness can be regulated to meet specific requirements [15,16]. These characteristics have led to numerous potential applications of hydrogels in the field of biomedicine, including drug delivery, hemostatic dressing, tissue engineering, biosensors, etc. [17,18]. Based on the sources of the polymers, hydrogels can be divided into natural and synthetic ones [19]. Natural hydrogels utilize natural polymers such as chitosan, collagen, hyaluronic acid, alginate, etc., which demonstrate exceptional biodegradability and biocompatibility as well as low immunogenicity [20,21,22]. Compared to natural ones, synthetic hydrogels originating from man-made polymers, such as polyethylene glycol, polyvinyl alcohol, polyacrylamide, etc., have better biostability, mechanical properties, and more tailorable structures [23,24]. To further improve the properties of hydrogels, it is common practice to synthesize hybrid hydrogels, which can be achieved through two different ways: (1) chemical modification of natural polymers, such as hyaluronic acid modified with histamine or methacrylate anhydride for improved gelation kinetics, mechanical strength, and structural stability [25,26]; or (2) the blending of natural and synthetic polymers, such as chitosan blended with polyvinyl alcohol or polyacrylic acid to obtain better porosity and swelling properties [27,28].

Injectable hydrogels are a special subclass of hydrogels, and their applications in insulin delivery have attracted considerable research attention in recent years [29,30,31]. Injectable hydrogels serving as insulin delivery systems offer many advantages, such as less frequent injections, bypassed first-pass metabolism, reduced risk of tissue infection, and localized insulin amyloidosis [32,33,34,35]. Depending on the working principle, injectable hydrogels can be categorized as in situ and shear-thinning ones. The liquid precursors of in situ hydrogels undergo gelation and form a polymeric network at the site of injection in response to factors such as the temperature, pH, and light [36]. Shear-thinning hydrogels transform from gel to sol as the crosslinking of the polymers breaks due to the shear force during injection, and the viscosity is recovered as the force is removed [37]. By developing injectable hydrogels with stimuli-responsiveness, the release of insulin can respond to various external stimuli, including the pH, temperature, ionic strength, etc., which enables precise control over insulin release that can mimic normal physiological rhythms, avoid a surge in the blood insulin concentration and minimize the risk of hypoglycemia [8,38].

**Figure 1:**
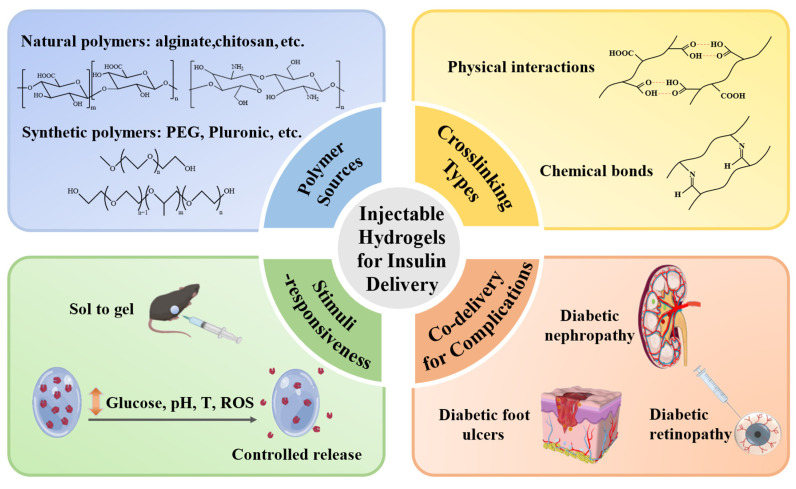
Injectable hydrogels for controlled insulin delivery to treat diabetes and its complications. Injectable hydrogels fabricated from natural or synthetic polymers and crosslinked via physical interactions or chemical covalent bonds can achieve stimuli-responsive insulin release for not only glycemic control but also treatment of complications such as nephropathy, skin ulcers, and retinopathy.

This review summarizes recent advances in injectable hydrogels for effective and controlled delivery of insulin, providing unique perspectives that go beyond glycemic regulation and encompass the treatment of diabetic complications (Figure 1). Detailed studies of the polymer sources and crosslinking mechanisms are synthesized to illustrate their effects on hydrogel performance and stimuli-responsive strategies (glucose, pH, temperature, ROS) to achieve dynamic glycemic control are highlighted. This review also incorporates forward-looking approaches involving the co-delivery of insulin with other therapeutics to manage diabetic complications. Although significant progress has been achieved, key challenges remain, including burst release, long-term biocompatibility, and scalability for clinical translation. Addressing these challenges will require further design optimization supported by rigorous experimental validation.

## 2. Polymer Sources for Preparing Injectable Hydrogels for Insulin Delivery

Natural and synthetic polymers are utilized to formulate injectable hydrogels for insulin delivery. Natural polymers offer inherent biocompatibility, biodegradability, and low toxicity, which help minimize adverse immunological responses and facilitate favorable interactions with biological tissues. Mechanical stability is the major limitation of natural polymers, restricting their use in long-acting delivery systems [39]. Synthetic polymers provide precise tunability, allowing the design of hydrogels with tailored properties for specific requirements. However, their relatively limited safety and biocompatibility necessitate careful evaluation to avoid undesirable in vivo responses. Striking a balance between degradation rates and mechanical properties is critical for achieving long-term efficacy with favorable biosafety. The system should function as a stable in situ depot offering controllable insulin release without inducing hypoglycemia and gradually degrade without causing long-term toxicity or immunological rejection. Emerging strategies increasingly focus on developing hydrogels with hybrid sources that combine both advantages to enhance functionality and safety through various modification approaches.

### 2.1. Natural Polymers

Natural-polymer-based hydrogels have found widespread applications in drug delivery due to their good sustainability, biocompatibility, and biodegradability [40]. The characteristics of commonly used natural polymers, including chitosan, alginate, guar gum, cellulose, and silk fibroin, for fabricating injectable hydrogels for insulin delivery are summarized in Table 1. However, hydrogels composed of only natural polymers have certain limitations regarding their mechanical properties and biostability, which restrict their practical utility, especially in scenarios where hydrogels are required to withstand external forces and ensure long-lasting durability for prolonged drug delivery [41,42,43]. Strategies to improve the properties of natural hydrogels include crosslinking and grafting with other monomers, and blending with synthetic polymers [44]. In the following part, the natural polymers, including polysaccharides and proteins, used in recent years for the preparation of injectable hydrogels for insulin delivery and their corresponding modifications will be described in detail.

#### 2.1.1. Polysaccharides

Chitosan is a cationic aminoglycan derived from the alkaline deacetylation of chitin [54]. It is widely applied in the area of biomedicine owing to its inherent pharmacological capacities, such as its hemostatic, antibacterial, anti-inflammatory, and anticarcinogenic properties, as well as its outstanding biocompatibility and biodegradability [45,55,56]. The cavity structures of chitosan-based hydrogels allow the effective loading of drugs, demonstrating excellent drug delivery capacity [57]. Zhang et al. synthesized an injectable hydrogel based on phenylboronic-acid-modified chitosan, which exhibited a continuous porous structure conducive to the encapsulation and release of insulin. The prepared hydrogel was able to maintain its structure in the high-glucose environment (300 mg/dL) for up to 16 d, and sustained insulin release was observed for 36 h in vitro. The hydrogel extracts even resulted in the higher cell viability of human skin fibroblasts after coculture, which demonstrated its outstanding biocompatibility [58]. Another modification method was proposed by Damiri et al., in which chitosan was crosslinked with 4-formylphenylboronic acid via Schiff base linkage. The hydrogel possessed a strong swelling capacity, achieving a 90.02% encapsulation efficiency for insulin [59].

Alginate is a natural polysaccharide extracted from brown algae, serving as a suitable drug carrier since its gelation can be carried out under mild conditions by adding divalent cations such as Ca^2+^ [47]. As alginate is negatively charged, the properties and swelling behavior of alginate-based hydrogels are influenced by the pH conditions. Alginate solution thickens under neutral conditions, which contributes to the formation of in situ hydrogels [60]. Partially oxidizing the alginate chains to manipulate the biodegradability of alginate-based hydrogel is an effective approach to control the release rate of encapsulated drugs [61]. Volpatti et al. utilized sodium periodate to oxidize alginate, synthesizing polymers with oxidation extents of 0%, 2.5%, 5%, and 7%. The alginate with a higher oxidation extent presented more aldehyde functional groups on the polymer chains, which contributed to the formation of cyclic hemiacetals that can be readily hydrolyzed. It was observed that the hydrogels prepared from alginate with a higher oxidation extent showed enhanced biocompatibility and biodegradability, as more cellular infiltration occurred at the injection site of mice after 31 d, along with a more significant decrease in the implant’s volume. The insulin-encapsulated hydrogels synthesized from 2.5% oxidized alginate demonstrated optimal in vivo glycemic control, in which the blood glucose levels (BGLs) of diabetic mice were reduced to the normal range within 1.5 h and maintained for 10 d [62].

**Figure 2:**
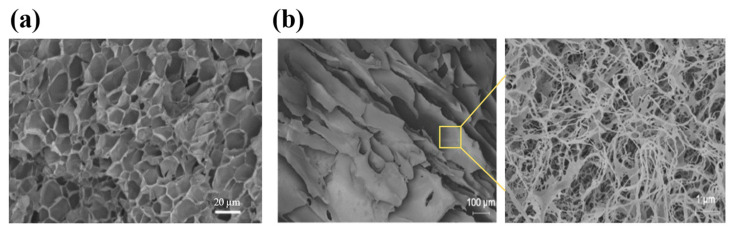
The structures of injectable hydrogels prepared with natural polymers, including polysaccharides and proteins. (**a**) A scanning electron microscopy (SEM) image of the guar-gum-based hydrogel. The hydrogel showed a large number of pores with thin pore walls, demonstrating the tightly crosslinked polymer network. (**b**) A field emission scanning electron microscopy (FESEM) image of the silk-fibroin-based hydrogel. A layer of silk fibroin nanofibers was formed and tightly covered the pores. Reproduced with permission from [65] Copyright 2021, Wiley-VCH GmbH (**a**) and [76] Copyright 2020, American Chemical Society (**b**).

Guar gum is a non-ionic polysaccharide extracted from the seed of *Cyamopsis tetragonolobus*, which is often used as a thickener in the food, pharmaceutical, and cosmetic industries [63]. Guar gum can form a highly viscous colloidal solution and exhibit shear-thinning properties. However, the high hydrophilicity of guar gum can cause the hydrogel to rupture due to excessive swelling, resulting in the rapid release of the loaded drugs. Therefore, guar gum is often crosslinked with other polymers to improve its mechanical properties and achieve prolonged drug release [64]. Lu et al. developed a guar-gum-based hydrogel (Figure 2a) in which guar gum was crosslinked with 4-carboxy-3-fluorophenylboronic-grafted chitooligosaccharides through phenylboronate ester bonds. The hydrogel displayed moderate strength and rigidity, with the viscosity decreased to 10 Pa·s when the shear rate exceeded 50 s^−1^, which indicated that the hydrogel can be readily injected with a common type of syringe. The in vitro release profile revealed an initial burst of insulin within 2 h, followed by a sustained release for 10 h [65].

Cellulose is a widely distributed polysaccharide with favorable biocompatibility, biodegradability, and mechanical strength, which is considered one of the major chemical resources for the future [66,67]. Due to the extensive presence of hydroxyl groups, cellulose molecules easily form networks through hydrogen bonds, but the large number of hydrogen bonds makes cellulose hardly soluble in water, posing a challenge for the preparation of hydrogels with cellulose [68,69]. To overcome such a problem, several water-soluble cellulose derivatives have been synthesized, one of which is hydroxypropyl methylcellulose (HPMC) [70]. HPMC is a widely used matrix for oral controlled-release tablets as it can form a gel in the water that serves as the barrier layer for drug release. The aqueous solution of HPMC can undergo a reversibly thermos-responsive sol–gel transition at 50–90 °C, which is an ideal property for constructing in situ injectable hydrogels [71,72]. To achieve an appropriate gelation temperature and improve the injectability, Okubo et al. constructed hydrophobically modified HPMC/cyclodextrin (HM-HPMC/CD) hydrogel by introducing stearyl groups to the hydroxypropyl ends of HPMC and blending with β-CD. As the hydrophobic moiety of HM-HPMC partially bonded to β-CD, the intermolecular interactions of HM-HPMC were inhibited and therefore HM-HPMC/CD formed a low viscosity sol. When the temperature rose, the dissociation of CD resulted in increased viscosity and gelation occurring at body temperature. Insulin-encapsulated HM-HPMC/CD hydrogel can be readily injected in mice by using a syringe with a 27 G needle, leading to the in situ gelation that formed a depot for sustained glycemic control lasting 12 h [73]. Earlier studies have also investigated other cellulose derivatives, including methylcellulose (MC) and carboxymethylcellulose (CMC) [74,75]. Overall, these derivatives have attracted significant research interest owing to their ability to form viscous solutions, retain water effectively, and respond to stimuli.

#### 2.1.2. Proteins

Silk fibroin is a natural protein obtained from silkworms, exhibiting excellent biocompatibility and exceptional mechanical properties, such as strength and toughness, that outperform many natural and synthetic polymers [77]. The mechanical properties and biostability of silk-fibroin-based biomaterials are closely related to the quantity, distribution, and orientation of the β-sheet structures [78,79,80]. Silk fibroin hydrogels are widely used for drug delivery, in which the β-sheets can act as barriers to drug diffusion, allowing a gradual release of the encapsulated drugs from the semi-amorphous regions of random coils between the β-sheets [81,82]. However, due to the slow transition rate from random coil to β-sheets, the gelation of silk fibroin needs a rather long time (4 days) in aqueous media, which limits its application [15,52]. Maity et al. have developed two approaches to address this issue by adding viscous agents and introducing chemical crosslinking separately. The addition of ethylene glycol and triethylene glycerol increased the medium viscosity and thus restricted the rotation of emerging β-sheets, which led to the formation of silk fibroin hydrogel in 50 min. The hydrogel exhibited a mesoporous structure, as shown in Figure 2b, which allowed the loading of insulin and retained its functional conformation. The normoglycemia of diabetic rats was maintained for 4 d after the hydrogel was injected subcutaneously [76]. In another study, a phenylboronate ester was formed between the boronic acid group on phenylboronic acid and the serine residues of silk fibroin. The modified silk fibroin was synergistically crosslinked by the phenylboronate ester and β-sheet, and the hydrogel could readily form in 30–60 min at 37 °C. Controlled release of insulin from the hydrogel managed to restore the glucose level of type 1 diabetic Wistar rats to physiological conditions for 36 h [83].

### 2.2. Synthetic Polymers

Despite potential issues such as the inflammatory response, in vivo accumulation, and toxic by-products of degradation, the highly tunable molecular weight and structure of synthetic polymers allow for easy modification of hydrogel properties such as the gelation kinetics, swelling behavior, mechanical strength, biostability, and stimuli-responsiveness [84]. Synthetic polymers generally show enhanced pharmacokinetics and longer drug release durations, indicating their promising potential in the field of drug delivery [85,86]. Several synthetic polymers that are commonly used for the fabrication of injectable hydrogel as insulin carriers will be introduced in the following sections.

#### 2.2.1. Polyvinyl Alcohol (PVA)

PVA is synthesized by the free radical polymerization of vinyl acetate followed by partial hydrolysis. It is a synthetic polymer with a large production yield, hydrophilicity, biocompatibility, and biodegradability [87]. With good water absorption and mechanical properties, PVA hydrogels are used as drug carriers that can increase the solubility and stability of drugs, but the drug release rate is relatively fast, depending on the crosslinking mechanism [88,89,90]. Ali et al. explored the effects of pinacolato ester bonds that formed between PVA and bisboronic acids on the mechanical properties and insulin release behavior of PVA hydrogel. It has been found that PVA hydrogel crosslinked by 1,4-benzene-diboronic acid bis(pinacol) ester (L1) exhibited a rapid shear-thinning behavior due to the predominant interchain crosslinking, and the in vitro insulin release from L1-PVA hydrogel after 4 h was less than 20%. In contrast, the PVA hydrogel crosslinked by bis[(pinacolato)boryl]methane (L4) demonstrated rapid insulin release followed by sustained release under the same condition (100 mM glucose), and the cumulative release reached over 75% in 3 h. This was because the crosslinking of the PVA-L4 hydrogel was intrachain-dominated, leading to the formation of a soft matrix. The study found that the tunable viscoelasticity of PVA-based hydrogels was associated with the composition of the intermolecular and intramolecular crosslinking, which represented an important strategy for constructing injectable PVA-based hydrogels for insulin delivery [91]. In a separate study, Ali et al. fabricated chitosan nanoparticle–PVA hydrogels (CPHGs) using various formylphenylboronic acid (FPBA) derivatives. All the CPHGs exhibited elastic solid-like behavior and enhanced water-holding capacity, making them suitable for encapsulating water-soluble therapeutic agents. CPHGs crosslinked with 4-FPBA or 2-fluoro-4-FPBA demonstrated high mechanical strength and slow insulin release attributable to their tight crosslinking, with less than 50% over 24 h. In contrast, the lower crosslinking density of 3-FPBA resulted in favorable self-healing and shear-thinning properties, allowing more rapid glucose-responsive delivery. Administration of 3-FPBA-crosslinked CPHGs loaded with 20 IU/kg of insulin significantly reduced the BGLs of mice within 4 h, with no burst release and maintenance of normoglycemia for 24 h [92].

#### 2.2.2. Poly(N-Isopropylacrylamide) (PNIPAM)

**Figure 3:**
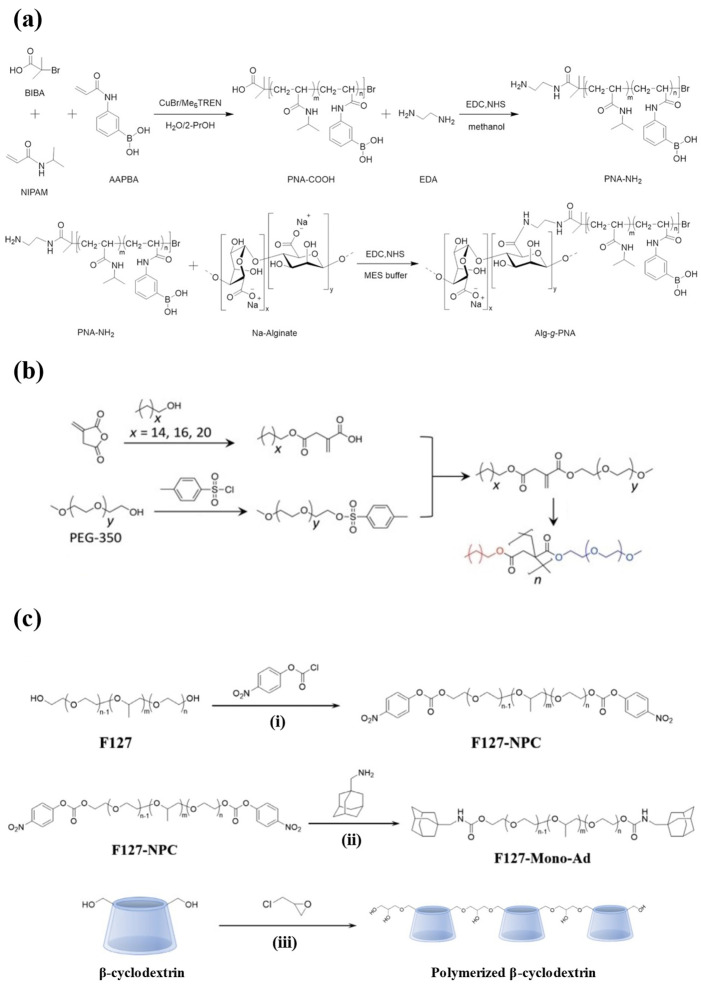
The synthesis schemes for injectable hydrogels prepared from synthetic polymers. (**a**) The synthesis routes of the PNIPAM-based hydrogel. N-isopropylacrylamide (NIPAM) and 3-acrylamidophenylboronic acid (AAPBA) were copolymerized and then grafted with alginate. (**b**) The synthesis route of amphiphilic polymers from PEG, enabling self-assembly into the hydrogel. (**c**) The synthesis routes of PF127-Ad ((i) and (ii)) and CDP (iii). Reproduced with permission from [97] Copyright 2021, American Chemical Society (**a**), [98] Copyright 2022, Wiley-VCH GmbH (**b**), and [99] Copyright 2021, Elsevier (**c**).

PNIPAM is a thermo-responsive homopolymer with amphiphilicity [93]. The lower critical solution temperature (LCST) of PNIPAM hydrogel is 32 °C [94]. Beyond the LCST, the enhanced hydrophobic interaction results in deswelling, and thus the hydrogel undergoes a volume phase transition. This transition close to body temperature has drawn attention to the application of PNIPAM hydrogel in drug delivery [95]. However, PNIPAM hydrogels are not biodegradable, which limits their use as drug carriers in vivo [96]. Hu et al. grafted chitosan onto the PNIPAM copolymer chains via amide bonds (Figure 3a) to improve the biodegradability of the hydrogel, and the enhanced hydrophilicity led to gelation at physiological temperature. Below 25 °C, the hydrogel remained in a sol state and then rapidly transformed to gel after subcutaneous injection, effectively locking insulin and achieving sustained release over 50 h in vitro. Since the amine crosslinking can be hydrolyzed by peptidase in vivo, the hydrogel had good biodegradability, and the molecular weight of the degraded by-products was much smaller than the cutoff of renal clearance [97].

#### 2.2.3. Polyethylene Glycol (PEG)

PEG is a non-toxic polymer with the ability to resist recognition by the immune system [100]. The molecular weight of PEG polymer impacts the drug-loading capacity and release kinetics of hydrogels as drug carriers, and introducing hydrophobic segments into the structure of hydrophilic PEG has become an important means of adjusting the properties of hydrogels recently [101,102]. Dhayani et al. developed a PEG350 (molecular weight)-based hydrogel (Figure 3b) crosslinked via hydrophobic interaction with the hydrophobic hexadecyl alkyl chain. The hydrogel showed reversible shear-thinning properties by undergoing a gel–sol transition during injection and reconverting to gel in vivo. It was completely degraded in mice after 7 d without evoking an immune response. In addition, a single injection of the hydrogel into diabetic mice can simulate the effect of glycemic control achieved with four subcutaneous injections of insulin [98].

#### 2.2.4. Polyurethanes (PUs)

Polyurethanes (PUs) are rubbery materials with favorable mechanical properties featuring a transition temperature below room temperature [103]. PUs exhibit good biocompatibility, making them suitable for applications in implants and medical devices. However, limited biodegradability constrains their use in injectable hydrogels for insulin delivery. To address this, a commonly adopted strategy is the incorporation of hydrolyzable segments into the PU backbone, which enhances the water absorption and improves both the gelation and drug release kinetics of PU-based hydrogels [104,105]. Mandal et al. developed a cyclodextrin (CD)-extended polyurethane (PU-co-CD) hydrogel that significantly prolonged the insulin release and improved the biocompatibility. CD that features a lipophilic inner cavity and a hydrophilic outer cavity can form abundant hydrogen bonds with PU, improving the mechanical strength of the gel. The incorporation of carboxymethyl cellulose (CMC) further optimized the hydrophilic–hydrophobic balance, yielding stable shear-thinning behavior and a smooth sol-to-gel transition at physiological temperature. The extensive weak physical interactions between insulin and the PU-co-CD system coupled with CMC as a diffusion barrier sustained delivery, with 38% insulin released at 12 h in vitro. PU-co-CD/CMC hydrogel loaded with 2 IU/kg insulin was able to maintain the standard BGLs of mice for 3 d without triggering immune rejection [106].

#### 2.2.5. Block Copolymers

Block copolymers are macromolecules that contain chemically distinct polymer blocks linked in a linear or branched structure. The molecular architectures include diblock, triblock, multiblock, starblock, etc. [107,108]. Their properties, including biocompatibility, mechanical strength, thermal gelation behavior, drug release kinetics, etc., can be adjusted by the incorporation of different blocks with varied properties [109,110]. More importantly, many block copolymers show amphiphilicity, in which hydrophobic cargos can be stabilized in hydrophobic regions while hydrophilic segments are compatible with body fluids. This contributes to the improved pharmacokinetics and biocompatibility of drug carriers based on block copolymers [111]. Many block copolymers have been utilized to fabricate hydrogels for insulin delivery with improved bioavailability. Table 2 provides an overview of the properties of the synthetic polymers commonly used to build these block copolymers.

Among the various block copolymers, Pluronics is one of the most widely studied triblock copolymers, comprising poly(propylene oxide) (PPO) as the hydrophobic block and poly(ethylene oxide) (PEO) as the hydrophilic block, arranged in the sequence of PEO–PPO–PEO [112]. It is a thermo-responsive material that undergoes gelation at a temperature close to body temperature [113]. The varying proportions of PEO and PPO divide Pluronics into different models. Pluronic F127 (PF127) is a specific type of Pluronics that contains 70% PEO blocks and stands as one of the only two biocompatible Pluronics approved by the FDA [114]. PF127 is non-toxic and stable, and the preparation of PF127 hydrogel does not require the use of organic solvents. However, unmodified PF127 hydrogel can readily dissolve in body fluid, which serves as the major drawback when being used as a drug carrier due to the risks of drug leakage and burst release [115]. Lee et al. constructed a hydrogel delivery system by introducing adamantane (Ad) as the host molecule at the end of the PF127 chains, which then formed micelles and crosslinked with polymerized β-cyclodextrin (CDP) (Figure 3c). The system contained several physical interactions, including hydrogen bonds between PF127 monomers, hydrophobic interactions between PF127-Ad micelles, and host–guest interactions between micelles and CDP. These interactions allowed the micelles to tightly aggregate, so the biostability of the hydrogel system was enhanced. After subcutaneous injection, the hydrogel was maintained in mice for 30 d. Moreover, since β-cyclodextrin could form the host–guest complex with insulin, PF127-Ad-CDP hydrogel allowed the sustained and slow release of insulin in vitro for 80 d [99].

In addition to Pluronics, other specifically designed block copolymers, such as poly(lactide)-b-poly(ethylene glycol)-b-poly(lactide) (PLA-PEG-PLA), poly(lactide-co-glycolide)-b-poly(ethylene glycol)-b-poly(lactide-co-glycolide) (PLGA-PEG-PLGA), poly(ε-caprolactone)-b-poly(ethylene glycol)-b-poly(ε-caprolactone) (PCL-PEG-PCL), are also utilized in preparing injectable hydrogels for insulin delivery [116,117,118]. Their properties and performances will be described, respectively, in Section 4 and Section 5.

**Table 2:** The properties of the synthetic polymers used to build block copolymers for injectable hydrogels for insulin delivery.

**Synthetic Polymers**	**Hydrophi** **li** **city/Hydrophobicity**	**Properties**	**Refs**
Poly(ethylene glycol) (PEG)	Hydrophilic	Biocompatible; low immunogenicity	[119]
Poly(ethylene oxide) (PEO)		Biocompatible; bio-inert	[120]
Poly(lactic acid) (PLA)	Hydrophobic	Bioabsorbable; non-toxic; high strength	[121]
Poly(lactic-co-glycolic acid) (PLGA)		Formed from PLA blended with glycolic acid; properties similar to PLA but with lower cost and tunable degradation rate	[122]
Poly(caprolactone) (PCL)		Relatively biostable; low biocompatibility; high mechanical strength; low drug release rate when serving as drug carrier	[123]
Poly(propylene oxide) (PPO)		Water solubility decreases as temperature increases; commonly utilized to fabricate the triblock copolymer Pluronics	[115]

## 3. Physical and Chemical Crosslinking in Injectable Hydrogels

**Figure 4:**
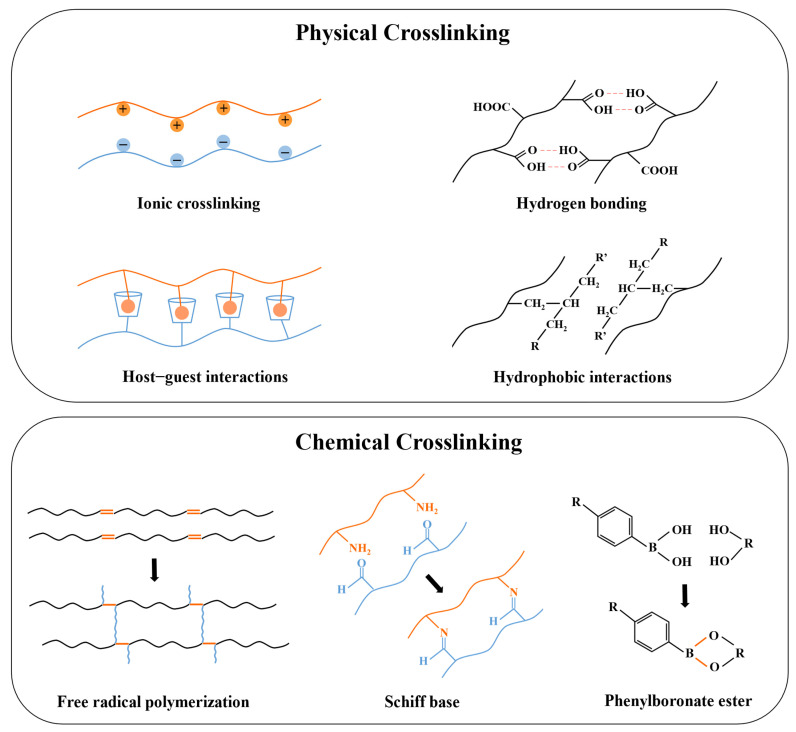
A schematic picture of physical and chemical crosslinking in injectable hydrogels prepared for insulin delivery.

Hydrogels are crosslinked via interactions between their polymer chains to maintain specific structures, which are significant for controlling their solubilities, mechanical properties, stimuli-responsiveness, drug-loading capacities, and drug-release kinetics [124,125,126]. As a subclass of hydrogel, the crosslinking used in ordinary hydrogels, such as hydrogen bonds, hydrophobic interactions, covalent bond formation via Michael-type addition and photopolymerization, etc., are also applicable to the injectable hydrogels, but the distinct requirements regarding the gelation kinetics, rheological properties, and release kinetics determine which one is more suitable [127,128,129]. The crosslinking in injectable hydrogels prepared for insulin delivery can be divided into physical and chemical ones (Figure 4).

### 3.1. Physical Crosslinking

Physical crosslinking involves non-covalent bonds, which avoid the cytotoxicity and the complex residue-removing procedures of chemical crosslinkers [130]. These relatively weak interactions will be broken when subjected to external forces, and the hydrogel exhibits shear-thinning properties which makes it suitable to be injected in vivo [131]. Common physical crosslinking involves hydrogen bonds, ionic crosslinking, hydrophobic interactions, host–guest interactions, and Van der Waals forces [34]. Hydrogen bonds and Van der Waals forces are both weak interactions, but combining these forces contributes to the formation of a more robust and stable network [132]. Maity et al. introduced Van der Waals forces into silk fibroin by adding glycols, resulting in tightly covered silk fibers in the laminar layer of the hydrogel [76]. Hydrophobic interactions can facilitate the rapid aggregation and self-assembly of hydrophobic groups in polymers when the temperature increases and exceeds the LCST, resulting in the formation of micelles that exhibit temperature sensitivity [133]. Therefore, polymer networks based on hydrophobic interaction, such as amphiphilic polymers, including PLGA-PEG-PLGA, PF127, etc., are often used to prepare injectable in situ hydrogels with thermal sensitivity [134,135].

In addition, hydrophobic interaction also contributes to the formation of host–guest interactions. Host–guest interaction utilizes molecules with cavity structures as the host to match the guest molecule with complementary shapes via physical interactions [136]. Cyclodextrin (CD) is one of the most commonly used host molecules possessing a hydrophobic inner cavity, which allows for hydrophobically binding with guest molecules. This strategy of forming a host–guest complex between CD and hydrophobic guests has been frequently adopted for different purposes. For example, Okubu et al. utilized the binding of β-CD and stearyl groups to enhance the injectability of HM-HPMC/CD hydrogel, while Lee et al. promoted the biostability and duration of insulin release of PF127 hydrogel by introducing β-CD to host adamantane (Ad) molecules conjugated at the terminal of PF127 [73,99]. Ionic-crosslinked hydrogels are constructed from oppositely charged molecules, which are sensitive to the pH and counter-ions [137]. Polysaccharide hydrogels are commonly synthesized via ionic crosslinking due to their natural ionizable moieties [138]. For instance, alginate can be readily crosslinked by divalent cations, Volpatti et al. reported that the acid-mediated insulin release from Ca^2+^-crosslinked alginate microgels was more rapid than from Ba^2+^-crosslinked ones, as the alginate microgels with Ba^2+^ crosslinking were less porous, resulting in reduced permeability [139].

### 3.2. Chemical Crosslinking

On the other hand, chemical crosslinking is achieved through the formation of covalent bonds and has stronger binding strength than physical interactions. Chemically crosslinked hydrogels present enhanced stability, mechanical strength, and highly consistent properties in vitro and in vivo, which allow for precise control of the pharmacokinetics [140,141]. Covalent interactions are generally irreversible, but certain dynamic covalent crosslinking, such as carbon–nitrogen double bonds (e.g., imine bond), boronate ester bonds, acetal bonds, etc., have been studied in depth since they are capable of being broken and reformed under specific temperatures, pHs, and ionic concentrations, conferring reversibility similar to physical crosslinking while maintaining the strength and stability of the covalent bond. These dynamic chemical crosslinkers have become an important strategy for designing stimuli-responsive hydrogels for drug delivery [142,143].

The main methods used to establish chemical crosslinking in injectable hydrogels include the chemical reaction of complementary moieties, free radical polymerization, Michael-type addition, and click chemistry [34]. However, the most common methods in the preparation of insulin delivery systems are via the formation of a Schiff base and phenylboronate ester, and free radical polymerization. Schiff base and phenylboronate ester bonds are extensively used due to their reversibility, which serves as an important strategy for injectable hydrogels to achieve controlled and precise insulin release. The Schiff base is the dynamic imine bond formed by the condensation reaction of amine and carbonyl groups. Due to being hydrolyzed in an acidic environment, it is extensively applied in the formulations of acid-responsive drug delivery systems [144]. The phenylboronate ester bond undergoes cleavage in a glucose-containing environment, and the exposed hydroxyl groups crosslink with the diol groups of glucose molecules. This results in the loosening of the polymer network, leading to the release of encapsulated insulin, and thus the hydrogels exhibit glucose-responsiveness [145]. These dynamic covalent bonds are extensively applied to construct stimuli-responsive systems, and the relevant studies will be reviewed in detail in Section 4.

Free radical polymerization is another method frequently utilized to generate chemical crosslinking in injectable hydrogels. Various types of initiators that respond to the temperature, light, and redox conditions are utilized to generate free radicals [146]. For the preparation of injectable hydrogels for insulin delivery, photopolymerization and redox polymerization are reported mostly. Photopolymerization requires the use of a photo-initiator and irradiation with light, and such polymerization has advantages, including rapid hydrogelation and tunable hydrogel properties [147]. Dhayani et al. fabricated a PEG-based injectable hydrogel by using, Irgacure 2959 as the photoinitiator and irradiating light at 365 nm, where a sustained release of insulin over 8 d in vitro was achieved with the prepared hydrogel [98]. In redox-initiating polymerization, an electron donor serves as the reductant to catalyze the decomposition of the oxidative initiator to generate free radicals at a relatively rapid and stable rate [148]. There are many combinations of redox initiators. The ones utilized frequently for the synthesis of injectable hydrogels for insulin delivery were ammonium persulfate (APS) and N, N, N′, N′-tetramethylethylenediamine (TEMED); for instance, Zhang et al. reported a PNIPAM-based hydrogel that can release insulin for 48 h [149].

## 4. Stimuli-Responsive Injectable Hydrogels for the Controlled Delivery of Insulin

To achieve the controlled release of insulin, different types of stimuli-responsive injectable hydrogels have been developed. Such kinds of systems form a depot in vivo after injection, allowing the release of encapsulated insulin in appropriate doses when needed to achieve more effective glycemic control and also to reduce the frequency of injection [150,151]. By incorporating stimuli-responsive polymers or units in the networks, the hydrogels undergo controlled swelling or shrinkage, which leads to the release of insulin in a regulated manner when receiving specific triggers [152,153]. The most commonly applied stimuli include glucose, pH, temperature, and reactive oxygen species (ROS) [154].

### 4.1. Glucose-Responsiveness

**Figure 5:**
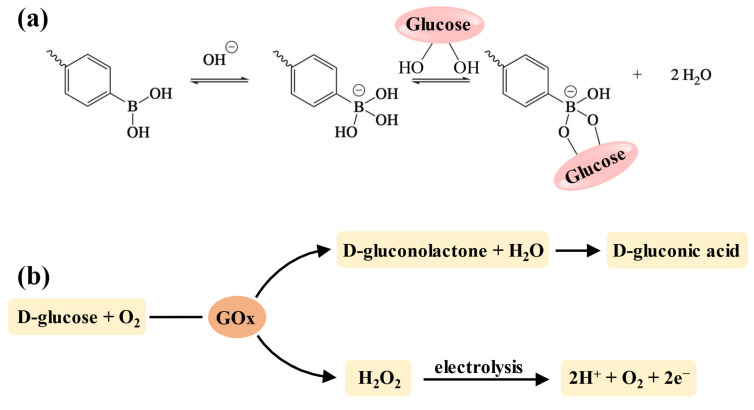
The mechanism of glucose-responsive units. (**a**) The mechanism of the PBA–diol structure for developing glucose-responsiveness. When pH ≥ pKa, PBA shifts to the negatively charged tetrahedral form with a higher affinity for glucose [145]. (**b**) The mechanism of GOx for developing glucose-responsiveness.

Among the various stimuli-responsive types, glucose-responsive delivery of insulin holds significant importance for blood glucose control since it can dynamically maintain normoglycemia and reduce the risk of hypoglycemia [155]. Currently, the strategy for developing glucose-sensitive hydrogels is to incorporate units that can interact with glucose into the crosslinking network, including concanavalin A (Con A), phenylboronic acid (PBA) (and its derivatives), and glucose oxidase (GOx) [156]. However, due to the hepatotoxicity of Con A, PBA (and its derivatives) and GOx are more widely used in injectable hydrogels for insulin delivery [157,158]. The boronic acid groups of PBA and its derivatives can specifically bind to the diol structures in the polymer chains to form the reversible phenylboronate ester bonds [159]. Thus, when the hydrogels are placed in a glucose-containing environment, glucose molecules with 1,2-cis-diols compete with those diol groups in the polymer chains to bind with the boronic acid groups (Figure 5a), resulting in the disruption of polymers due to broken crosslinking, and drug release is achieved accordingly [160]. Additionally, the chemical stability and biocompatibility of PBA make it suitable for long-acting insulin delivery without eliciting an immune response [158]. GOx is an endogenous catalyst that facilitates the oxidation of glucose into gluconic acid and H_2_O_2_, which further decomposes into protons and releases electrons (Figure 5b) [161,162]. This process enhances the electrostatic interactions in the hydrogel network, resulting in swelling, shrinkage, or degradation that allows for drug release [19].

**Figure 6:**
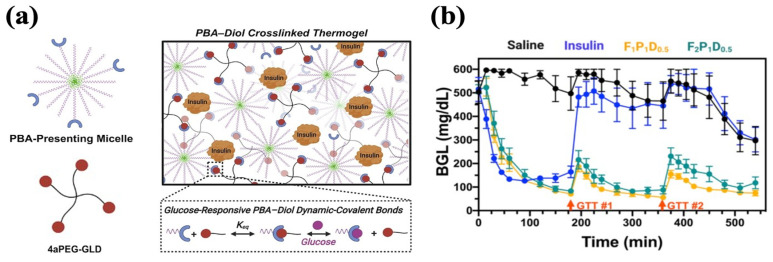
Glucose-responsive PF127-PBA hydrogel with PBA–diol crosslinking. (**a**) A schematic representation of PF127-PBA hydrogel. PBA-presenting Pluronic micelles were crosslinked with 4-arm PEG diol via dynamic phenylboronate ester bonds. (**b**) Glycemic control of PF127-PBA hydrogel in diabetic mice. GTT#1 and GTT#2 represent the intraperitoneal injection of glucose. Reproduced with permission from [135] Copyright 2022, American Chemical Society (**a**,**b**).

Many glucose-responsive injectable hydrogels have been established based on the above-mentioned mechanism. For example, Xian et al. end-modified Pluronic F127 (PF127) with PBA motifs (PF127-PBA) to develop an injectable hydrogel crosslinked by a tetravalent diol-modified four-arm PEG macromer (4aPEG-GLD). As the crosslinker, the binding of 4aPEG-GLD with PBA was broken by competition from free glucose, facilitating insulin release (Figure 6a). The hydrogels achieved the slow release of insulin in vitro, with 17% higher cumulative release in the 400 mg/dL glucose condition compared to the 0 mg/dL glucose condition, which demonstrated high glucose-responsiveness. A single treatment of hydrogel containing 6 IU/kg insulin conferred sustained glycemic stabilization over 9 h in diabetic mice. Within this interval, glucose tolerance tests (1.25 g/kg) performed at 3 and 6 h produced transient and attenuated spikes, followed by a return to normoglycemia each time (Figure 6b) [135].

Also based on the PBA–diol dynamic covalent bonds, Lv et al. utilized PBA–galactosyl covalent complexation to produce an injectable hydrogel–micelle composite for glucose-responsive delivery of insulin. In a high-glucose environment (5 g/L) in vitro, the insulin-loaded micelles exhibited the most rapid release, while the hydrogel matrix underwent severe degradation that enlarged the pore size to provide channels for insulin release from micelles. The composite system allowed 66.2% cumulative insulin release for 12 h under high glucose conditions and significantly slower insulin release of 17.7% under normal glucose conditions, which showed a release pattern similar to substantial insulin release in hyperglycemia and physiological insulin secretion in normoglycemia. The pattern was due to the utilization of different PBA derivatives, as micelles were crosslinked via 3-aminophenylboronic acid (APBA) and galactose, while the hydrogel matrix was crosslinked via 3-fluoro-4-carboxyphenylboronic acid (FCPBA) and galactose with stronger binding but less glucose sensitivity. At high glucose levels, the crosslinking of the micelles and hydrogel was broken to promote rapid insulin release. Under normal glucose conditions, only the crosslinking of micelles was disrupted and the hydrogel matrix acted as a diffusion barrier for minimal insulin release. The hydrogel–micelle composite maintained normoglycemia for approximately 24 h in vivo, with the BGLs returning to standard levels within 45 min after each of three glucose injections (3 mg/kg). No significant inflammation was observed at the injection site. The study highlighted a design approach where different PBA derivatives can be utilized together to develop the required glucose sensitivity in delivery systems to achieve the desired release rates of insulin [163].

**Figure 7:**
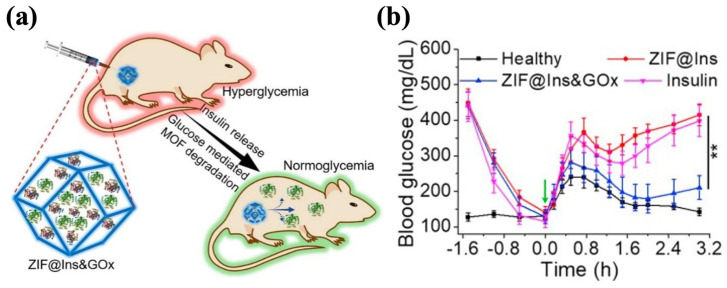
The ZIF@Ins&GOx-PF127 hydrogel. (**a**) A schematic picture of ZIF@Ins&GOx nanoparticles encapsulated in the PF127 hydrogel matrix. (**b**) In vivo glycemic control of injectable ZIF@Ins&GOx-PF127 hydrogel. The green arrow indicates intraperitoneal glucose injection. ∗∗ indicates *p* < 0.01. Reproduced with permission from [170] Copyright 2020, Elsevier (**a**,**b**).

GOx has the significant advantage of high specificity for glucose, but given its enzyme nature, its susceptibility to degradation and low activity pose challenges in realistic applications [164]. To overcome these limitations, zeolitic imidazole framework-8 (ZIF-8) has attracted attention and is emerging as a nanocarrier for drug storage and delivery [165]. ZIF-8 displays favorable biocompatibility and biodegradability, and its tunable properties, such as its crystalline structure, specific structure area, and cavity size, enable the effective loading of proteins while maintaining their activities [166,167,168,169]. In the local acid microenvironment resulting from glucose oxidation, the structure of ZIF-8 collapses to release the encapsulated insulin. Zhang et al. synthesized ZIF-8@Ins&GOx nanoparticles by encapsulating insulin and GOx in the cavities of ZIF-8. These nanoparticles were mixed with Pluronic F127 (PF127) to form the hydrogel, in which the ZIF@Ins&GOx nanoparticles were evenly dispersed in the matrix (Figure 7a). When administered at an insulin dose of 10 mg/kg, the ZIF-8@Ins&GOx-PF127 system maintained normoglycemia in diabetic mice for 3 d. The treated mice demonstrated glucose responsiveness comparable to healthy controls, decreasing to normal BGLs within 0.5 h of a glucose challenge (1.5 g/kg) and sustaining those levels for 2 h (Figure 7b). ZIF-8@Ins&GOx-PF127 demonstrated outstanding in vivo biosafety, showing no significant increase in the inflammatory cytokine levels within 7 d. Meanwhile, the hydrogel underwent gradual degradation, with 60% of the metal ions excreted in the treated group [170]. Similarly, Liu et al. prepared Ins@ZIF-8 nanoparticles and loaded them into the Poloxamer (another name for Pluronics)-based hydrogel that encapsulated GOx. The obtained Ins@ZIF-8/GOx-Gel showed a cumulative insulin release of 52% within 72 h under the 5 mM glucose condition, after which the release gradually ceased. Under high-glucose conditions (20 mM), the cumulative insulin release achieved 76% within 72 h, then escalated to 92% at 108 h. Such a pattern indicated that the release of insulin depended on the glucose levels. The BGLs of diabetic rats treated with Ins@ZIF-8/GOx-Gel (4 U/kg of insulin) were restored to standard levels after 3 h and remained stable for 7 h [171].

**Figure 8:**
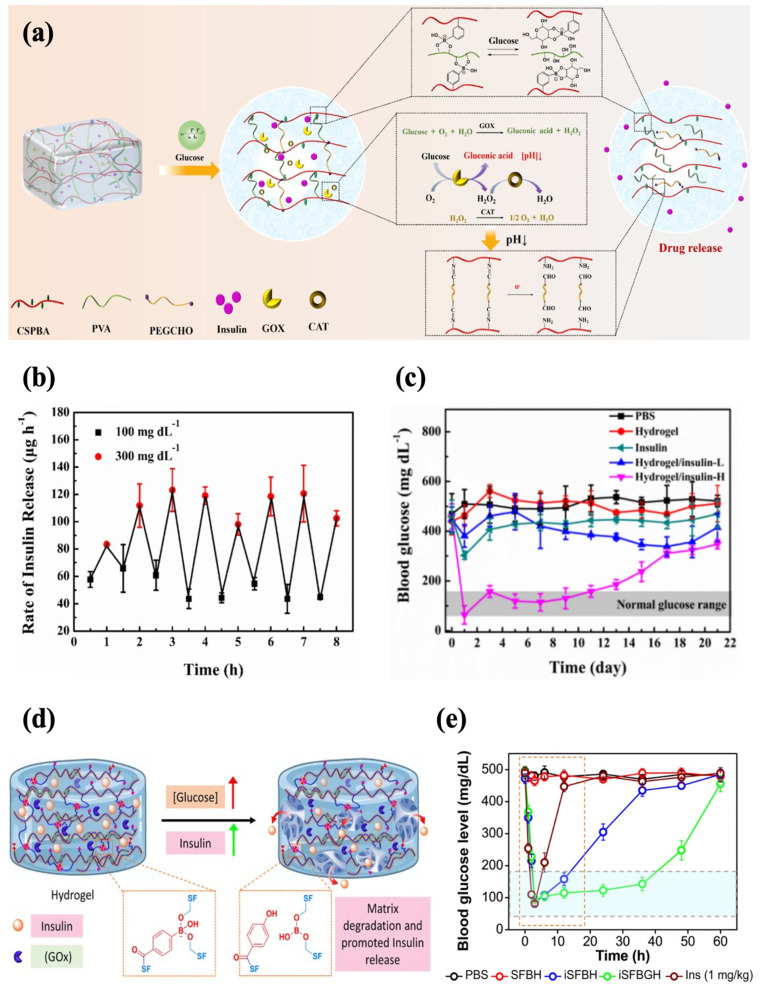
Glucose-responsive injectable hydrogel with both phenylboronate ester bonds and GOx as glucose sensors. (**a**) A schematic diagram of glucose-responsive chitosan-based hydrogel with dual sensors. (**b**) The profile of the insulin release rate of chitosan-based hydrogel at alternating glucose levels (100 mg/dL and 300 mg/dL). (**c**) The effect of chitosan-based hydrogel loaded with 0.6 wt% (H) insulin on the glycemic levels in diabetic mice. (**d**) A schematic diagram of glucose-responsive silk fibroin hydrogel with dual sensors. (**e**) The effect of silk fibroin hydrogel on the glycemic levels in diabetic mice (green line). Reproduced with permission from [58] Copyright 2023, Elsevier (**a**–**c**) and [83] Copyright 2023, American Chemical Society (**d**,**e**).

In addition, an injectable hydrogel with both PBA and GOx for enhanced sensitivity to glucose was also reported. Zhang et al. developed a glucose-responsive hydrogel with increased sensitivity based on GOx and phenylborate acid. The hydrogel network was crosslinked by Schiff base and phenylboronate ester bonds, and catalase (CAT) was introduced to ensure the activity of GOx as CAT facilitates the decomposition of H_2_O_2_ into oxygen and water. In a high-glucose environment, the phenylboronic ester bonds were broken to facilitate insulin release. Meanwhile, the degradation of H_2_O_2_ produced protons, which disrupted the Schiff base bonds and further promoted the release of insulin (Figure 8a). The formulation rapidly responded to alternating glucose levels in vitro, showing a typical pulsatile release pattern (Figure 8b). Subcutaneous injection of the hydrogel (0.6 wt% insulin) into diabetic mice enabled precisely controlled normoglycemia for 11 d, which displayed the capability for long-term glycemic control (Figure 8c). The hydrogel reversed hyperglycemia within 1 h following insulin administration (1.25 g/kg). It also showed good histocompatibility, with extensive degradation by 28 d and no evident tissue damage and inflammation [58]. In another work, GOx was encapsulated in PBA-modified silk fibroin hydrogel, with phenylboronate ester bonding formed between the boronic acid groups and serine residues (Figure 8d). The hydrogel (iSFBGH) incubated at a high glucose level (5 mg/mL) caused a sustained decrease in the pH of the medium over 24 h, indicating the retention of GOx activity. Meanwhile, the phenylboronate ester crosslinking broke slowly, which impeded the diffusion of GOx while providing a channel for insulin release. The well-maintained functional secondary conformation of the released insulin was affirmed. With the injection of iSFBGH containing 5 mg/kg insulin, the glycemic levels in diabetic rats were controlled within 2 h, and normoglycemia was sustained for 36 h (Figure 8e). The BGLs returned to normal within 1.5 h after a glucose challenge (2.5 g/kg), demonstrating robust glycemic regulation. The iSFBGH also exhibited low hemolytic activity and underwent complete degradation within 4 weeks post-injection [83].

### 4.2. pH-Responsiveness

**Figure 9:**
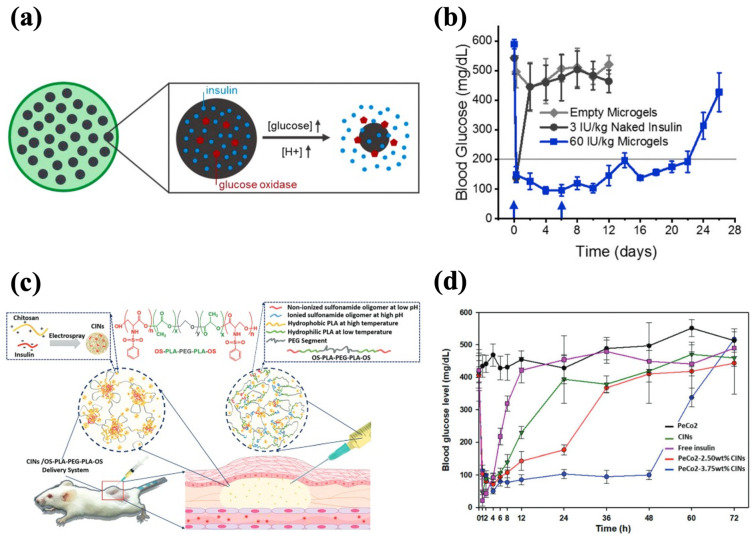
pH-responsive injectable hydrogel systems for insulin delivery. (**a**) A schematic diagram of NP-encapsulated alginate microgel and glucose-mediated pH-responsive insulin release. (**b**) The effect of microgel–NPs on the glycemic levels in diabetic mice. Blue arrows indicate subcutaneous injections of the 60 IU/kg microgel-NPs system at days 0 and 6. (**c**) A schematic diagram of CIN-encapsulated OS-b-PLA-b-PEG-b-PLA-b-OS hydrogel with OS being pH-sensitive modules. (**d**) The effect of PeCo2-CINs on the glycemic levels in diabetic mice. The PeCo2–CINs systems loaded with 3.75 wt% CINs demonstrated a sustained glycemic control effect. Reproduced with permission from [139] Copyright 2021, Elsevier (**a**,**b**) and [118] Copyright 2020, Royal Society of Chemistry (**c**,**d**).

pH-responsive hydrogels can modulate their structures based on the pH change, which ensures their adaptability in different physiological conditions and facilitates effective insulin release accordingly [172]. The construction of pH-responsive hydrogels can be achieved by introducing ionizable groups (e.g., hydroxyl, carboxyl), protonatable groups (e.g., amine), and pH-sensitive units (e.g., Schiff base) [86]. The incorporation of pH sensitivity into injectable hydrogels for insulin delivery can facilitate insulin release from GOx-based glucose-responsive hydrogels as well as modulate the gelation kinetics for long-acting delivery. Due to the hyperglycemia in diabetic patients, GOx can extensively convert glucose into gluconic acid, which results in a decreased environmental pH, and thereby the GOx-based hydrogels with pH-responsive units can undergo swelling, shrinkage, or degradation to accelerate insulin release [173]. In the studies of glucose-responsive hydrogels reviewed in the previous section, the Schiff base bonds and ZIF-8 framework that could be destructed at a low pH were incorporated into the GOx-based hydrogels to accelerate insulin release under high BGL conditions [58,170,171]. In addition, Volpatti et al. developed a system consisting of acetalated dextran nanoparticles (NPs) and alginate microgel, in which GOx and insulin were loaded. GOx induced the production of gluconic acid with a lower pH, resulting in the cleavage of the acetal bonds in the NPs for insulin release (Figure 9a). Encapsulation within the alginate microgel reduced the NPs’ diffusivity, retaining them at the injection site and extending insulin release. The microgel–NP system demonstrated long-term efficacy in diabetic mice, maintaining glycemic control for up to 3 weeks with only two treatments (Figure 9b) [139].

The pH-sensitive moieties can adjust the gelation kinetics of hydrogels, making the phase transition mainly dependent on the pH conditions [174]. It is also possible to establish hydrogel–insulin interactions through pH-sensitive moieties for prolonged delivery of insulin. For example, Nguyen et al. synthesized oligo-serine-b-poly(ε-caprolactone)-b-poly(ethylene glycol)-b-poly(ε-caprolactone)-b-oligo-serine (OS-b-PCL-b-PEG-b-PCL-b-OS) by conjugating serine sulfonamides to the end of PCL-b-PEG-b-PCL chains, with OS being the pH-responsive moiety. The sulfonamide groups in OS blocks could be deionized and become hydrophobic at physiological pH, leading to a sol-to-gel transition. The copolymer sols formed gels in situ and exhibited approximately 70% bioabsorption after 4 weeks, without causing abnormal symptoms at the injection sites in rats. To enhance the drug-loading efficiency and ensure even dispersion in the hydrogel, insulin-loaded chitosan nanospheres (CINs) were prepared and encapsulated in the hydrogel matrix, and the CIN-embedded hydrogel realized insulin release for 30 d in vitro. This prolonged delivery was achieved as the CINs carried a positive charge that could form electrostatic interactions with negatively charged OS blocks, contributing to the slow release of insulin [116]. Trinh et al. employed the same strategy to synthesize CINs and incorporated them into the OS-b-PLA-b-PEG-b-PLA-b-OS hydrogel (PeCo2–CINs system) (Figure 9c). The copolymer solution had strong fluidity below physiological pH and gel was rapidly formed at pH 7.4. A single administration of the PeCo2–CINs system produced a gradual decrease in the BGLs in vivo and steady control for 48 h (Figure 9d). The incorporation of CINs further slowed down the degradation of the hydrogel and improved its biocompatibility, resulting in approximately 30% mass retention in situ after 42 d [118].

### 4.3. Thermo-Responsiveness

Temperature is another widely applied trigger in insulin delivery systems as it can be readily accessed in vivo. In general, temperature-responsive hydrogels contain hydrophobic groups that cause the swelling or shrinkage of the hydrogel as a result of the temperature change. At high temperatures, the hydrophobic interactions between such groups are enhanced, leading to the dehydration and collapse of the hydrogel [175]. Therefore, one strategy to develop temperature-responsive hydrogels with ideal thermo-sensitivity involves adjusting the composition of the hydrophobic polymer chains [176]. Based on the thermo-responsive behavior, polymers can be distinguished into low critical solution temperature (LCST) and upper critical solution temperature (UCST) [177]. Among the injectable hydrogels, the LCST type is more commonly used, since they generally present as low-viscosity sols at room temperature and transform into gels after injection due to stimulation by the body temperature [38]. This characteristic helps prolong the retention time of insulin in situ and obviates the discomfort of injecting pre-formed hydrogels, which require greater shear force during injection [178].

Dutta et al. fabricated an injectable thermogel composed of PLGA–PEG–PLGA in which PLGA served as the hydrophobic block and PEG served as the hydrophilic block. This triblock copolymer could self-assemble into micelles at 4 °C and the sol–gel transition occurred at 34 °C. At 37 °C, the micelles aggregated due to the enhanced hydrophobic interactions caused by the dehydration of the PEG blocks, which led to the formation of a percolated network for drug release. The hydrogel was able to conduct sustained insulin release for 35 d in vitro while preserving the conformation [134]. In another study, Zhang et al. designed an injectable temperature-sensitive hydrogel (P(AAPBA-Dex-NIPAM)) intended for encapsulating insulinoma cells but also being able to load insulin. The network was formed by the crosslinking of poly(N-isopropylacrylamide (PNIPAM) as the temperature-responsive moiety with 3-acrylamidophenylboronic acid (AAPBA), and dextran-grafted maleic anhydride (Dex-Ma) served as the crosslinker. The sol–gel transition occurred at 28 °C, with an accelerated rate within 60 s at 37 °C. The in vitro release of insulin was characterized by a biphasic pattern, whereby 50% of the encapsulated insulin was rapidly released within the first 12 h, followed by a sustained release within 12–48 h, with a cumulative release of up to 80%. The hydrogel maintained the NIH3T3 cell viability above 80% after 48 h of co-incubation, meeting FDA guidelines for clinical application [149].

### 4.4. Reactive Oxygen Species (ROS)-Responsiveness

Diabetic foot ulcer is a severe complication caused by neuropathy that can lead to lower-extremity amputation [179]. Hyperglycemia induces the substantial production of ROS and chronic release of proinflammatory cytokines, resulting in prolonged and unhealable wounds [180]. Since insulin has been found to accelerate the proliferation of epithelial cells and the thickening of granulation tissue [181], an ROS-responsive hydrogel for the treatment of diabetic foot ulcers is also reported. Wang et al. proposed an ROS-responsive injectable hydrogel based on MnO_2_ nanosheets crosslinked with insulin-encapsulated aldehyde Pluronic F127 (PF127) micelles via the Schiff base. An MnO_2_ nanosheet, a novel nanoenzyme that catalyzes the generation of O_2_ from H_2_O_2_ (endogenous ROS), can promote wound healing by balancing ROS production and alleviating the hypoxic microenvironment. In this process, local accumulation of protons disrupted the Schiff base crosslinking of the matrix network, which in turn enabled insulin release. An in vitro study indicated that the release of insulin from hydrogel under ROS-enriched conditions (100 μM H_2_O_2_) was nearly twice that under control conditions, and the diabetic mice injected with the hydrogel exhibited more complete wound closure at 88.3% [12]. The study also demonstrated the potential of injectable hydrogels in delivering insulin to treat diabetic complications, and more related studies will be reviewed in Section 5.

### 4.5. Dual-Responsiveness

Dual-responsive hydrogels that respond to two different kinds of stimuli have gained increasing attention recently as they can provide a more precise release of insulin. Dual-responsiveness brings multiple advantages to insulin delivery systems. For instance, pH/temperature dual-responsive hydrogels allow more controls over the in situ forming of the depot since the sol–gel transition is triggered by the joint effects of the pH and temperature, and thus the gelation within the needle during injection can be inhibited [182,183]. Despite the shear-thinning property commonly demonstrated by glucose-responsive hydrogels based on PBA–diol, their injection still requires relatively large needle diameters and shear forces [29,184]. Thus, constructing a glucose/temperature dual-responsive hydrogel allows easy injection in the state of a highly fluidic sol and the subsequent formation of a gel in vivo, contributing to improved patient compliance [135]. The glucose/pH dual-responsiveness is a strategy for more precise control of release, particularly in glucose-responsive hydrogels based on GOx [185]. The catalytic action of GOx results in a lower pH, leading to the degradation or crosslinking breakage of the hydrogel, which further facilitates insulin release [58,186]. Typical examples of the dual-responsiveness of injectable hydrogels are listed in Table 3 and the relevant experimental results are summarized to demonstrate their potential in insulin delivery.

## 5. Injectable Hydrogels for the Co-Delivery of Insulin and Pharmaceuticals for the Treatment of Diabetes Complications

In addition to the skin ulcers mentioned earlier, the complications of diabetes mellitus also include retinopathy, nephropathy, and microangiopathy [187]. Diabetic retinopathy is a posterior segment eye disease that can cause blindness, but drug delivery to the posterior segment remains a challenge and many ocular delivery methods require frequent administration [188]. It has been found that insulin acceptors are expressed on the retina, enabling insulin to activate the vitality of retinal nerve cells through specific pathways [189,190]. To develop an extended ocular delivery system with the potential for retinopathy treatment, Rong et al. synthesized a hydrogel for subconjunctival injection using PLGA–PEG–PLGA as the matrix to encapsulate insulin nanoparticles. These insulin nanoparticles, with an average diameter of 137.5 nm, can pass through the blood–ocular barrier and reach the posterior segment. The hydrogel was able to conduct sustained insulin release for 60 d at a constant rate in vitro. The safety and biocompatibility were also confirmed, as subconjunctival injection of the hydrogel did not induce retinal ganglion cell death or retinal neuron damage, and the retinal ultrastructure remained intact [117].

**Figure 10:**
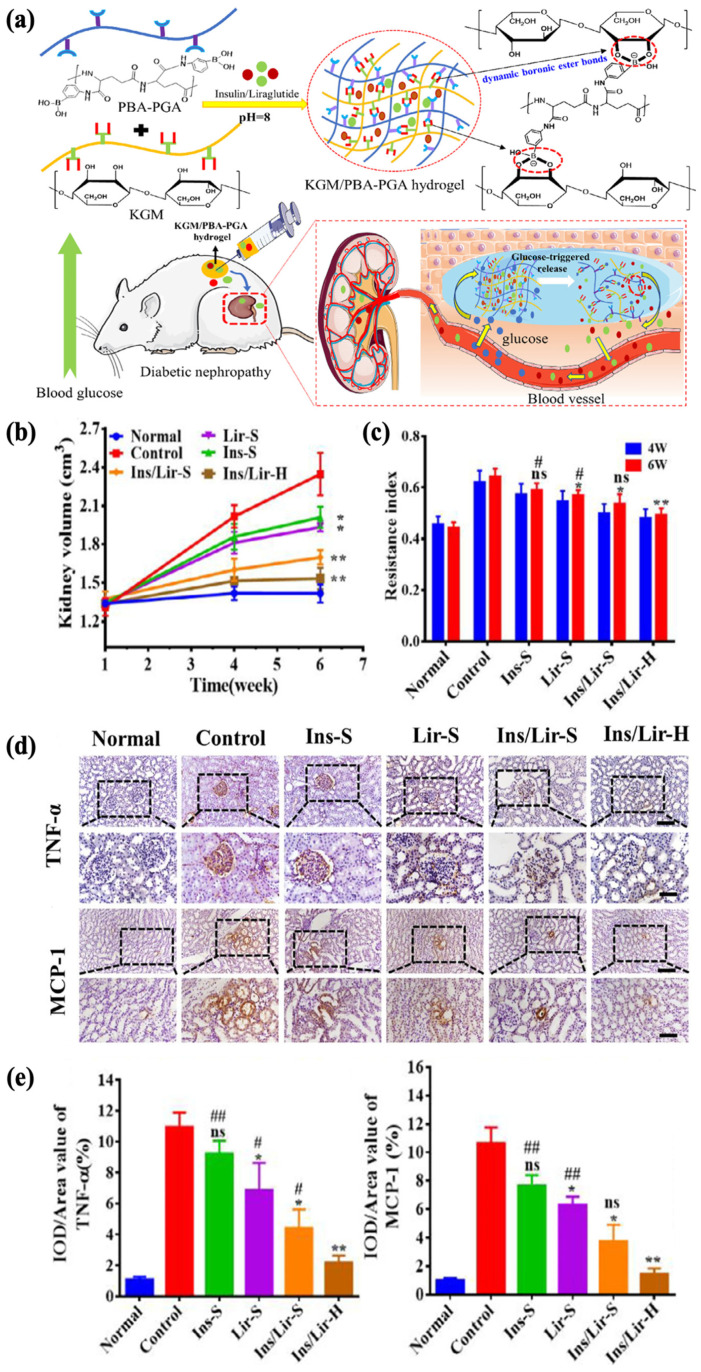
Glucose-responsive KGM/PBA–PGA hydrogel for the treatment of diabetic nephropathy. (* *p* < 0.05; ** *p* < 0.01, n = 3, compared with control group; # *p* < 0.05; ## *p* < 0.01, n = 3, compared with Ins/Lir-hydrogel group, ns, not significant) (**a**) A schematic of KGM/PBA–PGA hydrogel for the codelivery of insulin and liraglutide. (**b**) The kidney volume of diabetic rats. The renal hypertrophy of diabetic rats was relieved with Ins/Lir-H treatment. (**c**) The renal blood resistive index (RI) of diabetic mice. After 6 weeks of Ins/Lir-H treatment, the relatively low RI indicated the recovery of renal blood flow. (**d**) The immunohistochemical staining of renal TNF-α and MCP-1. (bar upper: 50 μm, bar below: 100 μm) (**e**) The quantitative statistics of TNF-α and MCP-1. The expressions of TNF-α and MCP-1 with Ins/Lir-H were reversed the most obvious. Reproduced with permission from [10] Copyright 2021, Elsevier (**a**–**e**).

Diabetic nephropathy, on the other hand, is another much more severe complication, with an incidence rate of more than 30% within 10 to 20 years after diagnosis [191,192]. Liraglutide is a receptor agonist for glucagon-like peptide-1, which regulates BGL by reducing glucagon secretion. Reports suggest its potential to improve kidney function without regulating the glycemic levels, and insulin in combination with liraglutide has been shown to significantly alleviate oxidative stress, which contributes to the mitigation of renal damage [193,194,195]. Tong et al. aimed to establish a delivery system for both insulin (Ins) and liraglutide (Lir) to control the progression of diabetic nephropathy. A glucose-responsive insulin- and liraglutide-co-encapsulated hydrogel (Ins/Lir-H) was synthesized through phenylboronate ester crosslinking between PBA-grafted γ-polyglutamic acid (PBA–PGA) and konjac glucomannan (KGM) (Figure 10a). The drug release behavior of Ins/Lir-H exhibited high sensitivity to glucose in vitro, in which pulsatile release was observed under alternating conditions of normal (1 mg/mL) and high (4 mg/mL) glucose levels. The KGM/PBA–PGA hydrogel had good biodegradability, retaining its structural integrity in rats for 3 d and completely degrading after 21 d, with no significant infiltrations of inflammatory cells at the injection region. After receiving hydrogel injection once every three days for six weeks, rats with diabetic nephropathy showed positive therapeutic results, including the inhibition of kidney enlargement (Figure 10b), increase in renal blood flow (Figure 10c), and reduction in the expression of inflammatory factors such as TNF-α and MCP-1 (Figure 10d,e). These results demonstrated the potential of KGM/PBA-PGA hydrogel as the carrier for the co-delivery insulin and liraglutide for treating kidney complications [10].

Diabetes is frequently accompanied by persistent inflammation and amplified oxidative stress. In type 2 diabetes, chronic low-grade inflammation contributes to β-cell death, further exacerbating insulin deficiency. Elevated levels of inflammatory markers such as C-reactive protein and interleukins are closely associated with diabetic microangiopathy [196,197]. A combination of insulin and anti-inflammatory agents has emerged as an important strategy to control the progression of type 2 diabetes. Heyns et al. conceptualized an injectable composite hydrogel based on chitosan and β-glycerol phosphate crosslinked via ionic interactions, which simultaneously encapsulated insulin and curcumin to mitigate chronic inflammation. Curcumin was encapsulated within PLGA nanoparticles to significantly enhance its solubility and bioavailability. Employing GOx as a glucose sensor, in vitro insulin release was accelerated under high-glucose conditions (500 mg/dL) and triggered on-demand at 300 mg/dL. This pulsatile behavior lasted for nearly 12 h. Meanwhile, curcumin was released in a sustained manner for up to 24 h. The composite system showed good biocompatibility, with no significant cytotoxic effects observed in FHs74 cells [198].

## 6. Conclusions and Outlook

Injectable hydrogels applied in the field of insulin delivery aim to alleviate patients’ discomfort resulting from frequent injections and provide sustained glycemic control. Depending on the polymers used, the hydrogels are classified into natural ones, including polysaccharides and proteins, and synthetic ones, including PVA, PNIPAM, PEG, and certain block copolymers. The combination of different polymers (hybrid ones) allows hydrogels to exhibit adjustable hydrophilicity, swelling behavior, structure, biocompatibility, and biodegradability. Polymer chains are crosslinked to form the hydrogel network through physical or chemical interactions, which is significant for the hydrogel properties, especially the mechanical strength and stimuli responsiveness. To adapt to changes in the physiological environment, stimuli-responsiveness is commonly developed for controlled delivery, and precise insulin release is achieved through the control of the temperature, glucose concentration, pH, and ROS. In addition to glycemic regulation, injectable hydrogels are also found to be promising for the treatment of diabetic complications, which expands its applications in diabetes treatment.

So far, no clinical trials or FDA-approved products related to injectable hydrogels for insulin delivery have been reported [199,200], suggesting that injectable hydrogels for insulin delivery are in the early stages of development and that more research is needed to validate their efficacy and in vivo safety. Endo’s Vantas^®^ has generated approximately USD 20 million in commercial value as an FDA-approved injectable hydrogel for the delivery of gonadotropin or luteinizing to control the progression of prostate cancer, demonstrating the potential economic returns of injectable hydrogels for delivering therapeutic agents [199]. Such a positive example provides more impetus for investment and research in this field, and the successful clinical translation of injectable hydrogels for insulin delivery is expected to offer a more convenient and effective treatment option for the vast diabetic population.

Early-stage laboratory studies are essential for proof of concept, but critical gaps remain. Longer-term in vivo studies extending over weeks are particularly necessary to confirm sustained efficacy and safety. Moreover, the degradation kinetics of these systems are not yet fully characterized. Short-term biocompatibility assays often fail to capture chronic adverse effects and ongoing tissue responses. Given that the material retention, clearance, and immunogenicity are directly linked to clinical translation, future investigations should monitor both the systemic and organ-specific impacts over extended periods and multiple administrations. Larger animal models, such as Göttingen minipigs, can enhance the translational relevance beyond rodent studies [201]. Currently, injectable hydrogels incorporating insulin or combination therapies for diabetic complications remain mostly conceptual. Demonstrating comprehensive therapeutic efficacy in established disease models is essential to move these approaches closer to clinical application.

A major challenge in developing injectable hydrogels for insulin delivery is mitigating the initial burst release, which risks hypoglycemia and rapid insulin depletion [32,202]. Some systems also exhibit limited loading capacity due to low-density crosslinking and low mechanical strength, which can fail to sustain clinically relevant doses of insulin for long periods [203]. A current approach involves developing dual polymer networks, such as interpenetrating networks [204]. By fine-tuning the network density, the mechanical stability and sustained release profile provided by a higher density with the enhanced stimuli-sensitivity and injectability of a lower density can be balanced, ultimately achieving optimal efficacy [205,206]. Meanwhile, 3D printing offers new avenues for customizing hydrogels with desirable properties, supporting sustainable bioengineering and personalized treatment [207,208]. Another emerging strategy is to exploit nanoparticles [209], particularly embedding nanoparticles within the hydrogel matrix to further regulate insulin diffusion. For example, Bayramia et al. developed an alginate-poly(3-hydroxybutyrate-co-3-hydroxyvalerate) PHBV nanoparticle hydrogel that significantly minimized the initial burst release (8% in 24 h) and prolonged insulin delivery (54.6% over 31 days) [210].

The major hurdles for the clinical translation of injectable hydrogels for insulin delivery include compliance with chemistry, manufacturing, and control (CMC) and good manufacturing practice (GMP) standards, as well as well-defined regulatory guidelines [200]. Although advanced hydrogel systems have been proposed, complex fabrication processes and multiple modifications can increase regulatory scrutiny and production costs. Adopting low-cost materials and simple, eco-friendly synthesis methods contributes to faster, more affordable product launches [211,212]. Currently produced on a small laboratory scale, the hydrogels must be integrated into large-scale manufacturing systems requiring GMP compliance. It is crucial to ensure batch stability, consistency, and reproducibility. For an in-depth analysis of the pathways to translation and industrial production, the following articles are recommended [213,214]. Beyond standard testing, evaluating hydrogel behavior and safety under abnormal physiological conditions is necessary to refine patient care and meet regulatory demands. In the future, multidisciplinary collaboration will be essential for designing injectable hydrogels with improved clinical utility.

## Figures and Tables

**Table 1:** Summary of the natural polymers utilized for the preparation of injectable hydrogels for insulin delivery.

**Natural Polymer**	**Functional Groups**	**Electrostatic Charge**	**Solubility**	**Advantages**	**Disadvantages**	**Refs**
Chitosan	-NH_2_, -OH	Positively charged at the acid condition	Soluble in acetic acid when pH < 4	Low cost; hemostatic, antibacterial, anti-inflammatory, and anticarcinogenic properties; outstanding biocompatibility and biodegradability	Ease of degradation	[45,46]
Alginate	-COOH, -OH	Negatively charged	Soluble in water	Low cost; rapid gelation under mild conditions by adding divalent cations (e.g., Ca^2+^)	Ease of degradation	[47,48]
Guar Gum	-OH	Neutral	Soluble in water	Low cost; anti-inflammatory activity	High viscosity for injection	[49]
Cellulose	-OH	Neutral	Insoluble in most aqueous and organic solvents	Favorable biocompatibility, biodegradability, and mechanical strength; biodurability	Low solubility; Plant sources require further purification	[50,51]
Silk Fibroin	-COOH, -NH_2_	Neutral	Soluble in water	Low cost; excellent biocompatibility; tunable biodegradability; superior mechanical strength; adaptability to multiple formats	Time-consuming gelation	[52,53]

**Table 3:** Representative examples of dual-responsive injectable hydrogels for insulin delivery.

**Dual-Responsiveness**	**Polymer**	**Biocompatibility**	**Insulin Loading Capacity (LC) or Encapsulation Efficiency (EE)**	**In Vitro Insulin Release (Duration and Cumulative Release Percentage)**	**Duration of Glycemic Control In Vivo After Single Injection**	**Ref**
Glucose and Temperature	Alginate-g-P(NIPAM-co-AAPBA)	Viability of L929 mouse fibroblasts remained at 100% after incubation for 24 h	Loading ratio 1.0 g/L	48 h; 70% at 5 g/L glucose condition (GC) and 30% at 1 g/L GC	/	[97]
	F127-PBA	Viability of C2C12 cells was maintained at over 95% after incubation for 24 h	Loading ratio 20 μg/100 μL	8 h; 36% at 0 mg/dL GC and 53% at 400 mg/dL GC	9 h in mice	[135]
	P(Lys-co-LysFCPBA)-b-PEG-b-P(Lys-co-LysFCPBA) & γ-P(GA-co-GAGal)	No inflammation at mice’s injection sites after 14 d	LC: 8.6 ± 0.4 wt%; EE: 13.0 ± 0.2 wt%	12 h; 62.6% at 5 g/L GC and 17.7% at 1 g/L GC	24 h in mice	[163]
Glucose and pH	CSPBA/PEGCHO/PVA/GOx	HSF cells exhibited higher viability, and injection sites on mice showed no inflammation after 4 weeks	LC: 0.3%	36 h; 16% at pH 7.4, and 70.2% at pH 6.5; 33% at 100 mg/dL GC and 51.8% at 300 mg/dL GC	11 d in mice	[58]
Temperature and pH	OS-b-PCL-b-PEG-b-PCL-b-OS	No abnormal symptoms at the injection site of mice after 1 month	LC: 20%; EE: 95.85%	30 d; Over 80% at pH 7.4	/	[116]
	OS-b-PLA-b-PEG-b-PLA-b-OS	The viability of 293 T and RAW 264.7 cells remained over 80% after incubation for 24 h	LC: 20%; EE: 96%	/	60 h in mice	[118]

## Data Availability

Data are contained within the article.
